# Phenolic Acid and Flavonoid Content Analysis with Antioxidant Activity Assessment in Chinese *C. pi. Shen* Honey

**DOI:** 10.3390/molecules30020370

**Published:** 2025-01-17

**Authors:** Ningxin Qi, Wen Zhao, Chenghua Xue, Lin Zhang, Han Hu, Yue Jin, Xiaofeng Xue, Rui Chen, Jinzhen Zhang

**Affiliations:** Bee Product Quality Supervision and Testing Center, Ministry of Agriculture and Rural Affairs, Institute of Apicultural Research, Chinese Academy of Agricultural Sciences, Beijing 100193, China

**Keywords:** phenolic compound, antioxidant activities, *C. pi. Shen* honey, different botanic origins

## Abstract

The nutritional value of honey is closely related to its components, which are highly influenced by the botanic origin. *C. pi. Shen* (*Codonopsis pilosula* (Franch.) var. modesta (*Nannf.*) L. T. Shen), a key plant in traditional Chinese medicine, mainly produced in Gansu Province of China, has attracted much attention for its medicinal value. However, there are few reports about *C. pi. Shen* honey. Therefore, this study aimed to evaluate the contents of phenolic profiles and antioxidant activities in *C. pi. Shen* honey by colorimetric, UPLC-MS/MS, Ferric ion Reducing Antioxidant Power (FRAP), 2,2-bisazo-bis (3-ethyl-benzothiazole-6-sulfonic acid) (ABTS) free radical capacity, and 1,1-diphenyl-2-trinitrophenylhydrazine (DPPH) scavenging ability methods. In comparison with four other high-yield unifloral honeys in China (*Acacia* honey, linden honey, rape honey, and jujube honey), *C. pi. Shen* honey demonstrated remarkable advantages. Specifically, the levels of total phenolic acids, total flavonoids, and phenolic components of *C. pi. Shen* honey were significantly pronounced, especially protocatechuic acid and kaempferol. Furthermore, the antioxidant activity of *C. pi. Shen* honey was also excellent, which was attributed to its phenolic compounds, underscoring its potential biological value. This study is anticipated to serve as a reference for the identification, nutritional assessment, and functional advancement of *C. pi. Shen* honey.

## 1. Introduction

Honey is defined as the natural sweet substance produced by bees (*Apis mellifera*) that is derived from nectar or insect excrement. Bees collect this substance in the hive for storage, incorporating their specific secretions, which are transformed and dehydrated until they are ripe [1]. Honey is mainly composed of glucose and fructose [2] and other components such as proteins, amino acids, phenolic compounds, enzymes, vitamins, minerals, etc. [3,4]. The composition of these components plays a significant role in the flavor characteristics and biological activities of honey [5].

Phenolic compounds are a class of secondary metabolites that are biosynthesized by plants and can be divided into phenolic acids and flavonoids (flavonols, flavones, flavanols, flavanones, anthocyanins, chalcones, isoflavones, etc.). Phenolic compounds are usually transferred from the plant to the nectar and subsequently to the honey by bees [6]. More than 160 phenolic compounds have been identified in honey, of which the most common are caffeic acid, gallic acid, hydroxybenzoic acid, quercetin, kaempferol, and apigenin [7,8]. These compounds are renowned for their multifaceted biological activities, encompassing antioxidant, antimicrobial, neuroprotective, and anticancer properties [9,10]. As biologically active substances, the composition and content of phenolic compounds in honey can be affected by factors such as plant source, geographical origin, or harvest time [11,12,13,14,15]. In addition, there are also differences in the content of phenolic compounds in the same honey variety [16,17,18]. Therefore, the analysis of the phenolic compounds of different honey types can help to identify varietal differences and elucidate potential biological value, thereby emphasizing individual differences.

Studies have shown that there is a correlation between the phenolic content of most honeys and the antioxidant activity they exhibit [19,20,21,22,23,24,25]. Phenolic compounds are frequently employed as free radical scavengers, metal chelating agents, or regulators of enzymatic and non-enzymatic systems, with the objective of controlling the redox balance of cells [26]. It has been reported that phenolic acids (such as *p*-coumaric acid, caffeic acid, protocatechuic acid, and gallic acid) have the property of resisting oxidative stress [27,28,29], while flavonoids in honey can protect lipids from oxidative damage to cell membranes [30]. The antioxidant activity of honey is generally measured using antioxidant assays approved for use in food and beverages, including the Ferric ion Reducing Antioxidant Power (FRAP), 2,2-bisazo-bis (3-ethyl-benzothiazole-6-sulfonic acid) (ABTS) free radical capacity, and 1,1-diphenyl-2-trinitrophenylhydrazine (DPPH) scavenging ability analysis methods [31]. Among these, the FRAP assay is a typical electron transfer-based method that measures the reduction of the ferric ion (Fe^3+^)–ligand complex to the intensely blue-colored ferrous (Fe^2+^) complex by antioxidants in an acidic medium; 2,2-bisazo-bis (3-ethyl-benzothiazole-6-sulfonic acid) (ABTS) free radical capacity tests are mainly based on the single electron transfer that occurs between the antioxidant and substrate; and the 1,1-diphenyl-2-trinitrophenylhydrazine (DPPH) scavenging ability method is mainly based on the analysis of electron or hydrogen atom transfer (mixed) [32].

In our previous research, honey from medicinal plants was found to have unique compositions and potentially functional components [33,34]. *Codonopsis*, a dried root from plants in the *Codonopsis* genus (Campanulaceae family), includes varieties such as *Codonopsis pilosula* (Franch.) *Nannf.*, *C. pi. Shen* (*Codonopsis pilosula* var. modesta *(Nannf.)* L. T. Shen), *Codonopsis tangshen* Oliv., and *Codonopsis tubulosa Kom*, which is recognized as one of the authentic medicinal materials in China and has garnered sustained interest and attention in recent years. There have been reports about phenolic compounds in the honey of *Codonopsis pilosula* (Franch.) *Nannf.* from Xinjiang [35] and Changbai Mountain (Jillin) [36] in China. However, *C. pi. Shen* honey, another *Codonopsis* characteristic honey variety, has received relatively little attention, and its characteristics remain unknown. Clarifying the phenolic composition and antioxidant activity of *C. pi. Shen* honey will help consumers better understand the characteristics of this special type of honey. It is well known that the origin of a plant may affect its composition and nutritional value. The quality of honey is also affected by the growing environment of its source plant. Zhouqu County in Gansu Province of China is the main planting area of *C. pi. Shen*. This area is located at an altitude of over 2000 m and provides a suitable climate and abundant nectar sources for the local production of *C. pi. Shen* honey. Therefore, the aim of this study was to evaluate the phenolic profile and antioxidant capacity of *C. pi. Shen* honey, while also referencing the results of four other high-yielding Chinese unifloral honeys (linden honey, *Acacia* honey, rape honey, and jujube honey). This study facilitates the accurate identification and evaluation of the quality of *C. pi. Shen* honey, thereby providing a foundation for the study of characteristic honey varieties.

## 2. Results and Discussion

### 2.1. Phenolic Compound Analysis

#### 2.1.1. Total Phenolic Acid and Total Flavonoid Content Analysis

**Figure 1:**
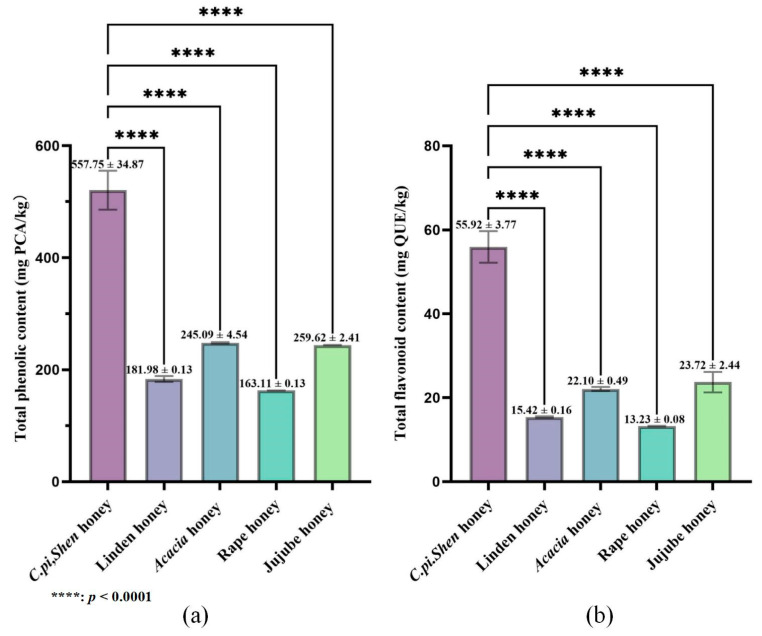
(**a**) Total phenolic acid and (**b**) total flavonoid contents of *C. pi. Shen* honey and four other kinds of honey.

The contents of total phenolic acids and total flavonoids of *C. pi. Shen* honey and four other kinds of honey were analyzed, and the results are shown in Figure 1. The results reveal that there are significant differences in the total phenolic acid and total flavonoid contents between the different types of honey (*p* < 0.05). The main cause of this phenomenon is most likely the differences in plant origin. A high content of total phenolic acids and total flavonoids has been reported in *C. pi. Shen* plants [37,38] due to the honey production process of bees, which may eventually affect the contents of the components in *C. pi. Shen* honey. Another reason may be related to the climate at the collection site [39]. Perhaps because *C. pi. Shen* plants tend to synthesize more phenolic substances under sufficient moisture conditions, there are more metabolites, such as phenolic acids, in *C. pi. Shen* nectar and, ultimately, the total phenolic acid and total flavonoid contents are higher in *C. pi. Shen* honey.

#### 2.1.2. Quantitative Analysis of 23 Phenolic Compounds

The results of the analysis of 23 common phenolic compounds in five types of honey are presented in Table 1. In comparing the five honeys in terms of the compounds detected, the main differences were found in the phenolic composition and the specific content.

**Figure 2:**
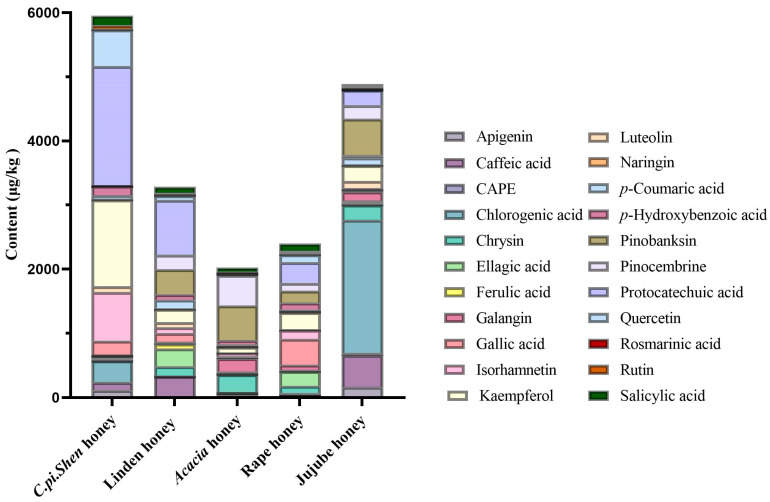
Distribution of phenolic compounds in *C. pi. Shen* honey and four other kinds of honey.

The phenolic compound composition of five honeys is shown in Figure 2. It can be seen that *C. pi. Shen* honey is rich in phenolic compounds, and its specific composition is different from that of the other four honeys. Further analysis using a significance test (as shown in Table 1) revealed that the contents of four phenolic compounds in *C. pi. Shen* honey were significantly higher than those in the other four honeys. These compounds include apigenin, isorhamnetin, kaempferol, and protocatechuic acid (*p* < 0.05). The mentioned phenolic compounds may be of great importance for the specific identification of honey.

The principal component identified in *C. pi. Shen* honey was protocatechuic acid, with a range of 1214.9 to 2496.2 µg/kg, which was significantly higher than that in other kinds of honey. The content of kaempferol in *C. pi. Shen* honey was the second-highest detected phenolic compound, ranging from 538.1 to 2169.8 µg/kg. This may be related to the abundance of flavanone glycosides in its nectar. During honey production by bees, glycosides are hydrolyzed to flavonoid aglycones (kaempferol) under the catalytic action of enzymes [40]. The phenolic metabolites of honey are also affected by altitude. Higher levels of quercetin and its metabolites, kaempferol glycosides, etc., are found in honey from high altitude areas (greater than 1700 m above sea level) [41]. As the production area of *C. pi. Shen* honey is located at an altitude of over 2000 m, this could be another reason for its high kaempferol content.

Table 2 compares the *C. pi. Shen* honey results of this study with those of previous studies on *Codonopsis* honey. Besides the difference in the number of compounds detected, the content of protocatechuic acid identified in *C. pi. Shen* honey was significantly higher than that previously reported for *Codonopsis* honey 1, in which the mean content was 96 µg/kg [35], while protocatechuic acid was not detected in *Codonopsis* honey 2. As for kaempferol, although the content of kaempferol in *Codonopsis* honey 2 was among the top two highest results, it was still significantly lower than the content of kaempferol in *C. pi. Shen* honey (*p* < 0.05), and the mean content of kaempferol in *Codonopsis* honey 1 was 14 µg/kg [35]. This demonstrates that even if the botanical origin of the honey is from the same genus, the contents of phenolic substances can still vary significantly due to factors such as different geographical sources and weather.

### 2.2. Antioxidant Activity Analysis

#### 2.2.1. FRAP Assay Analysis

**Figure 3:**
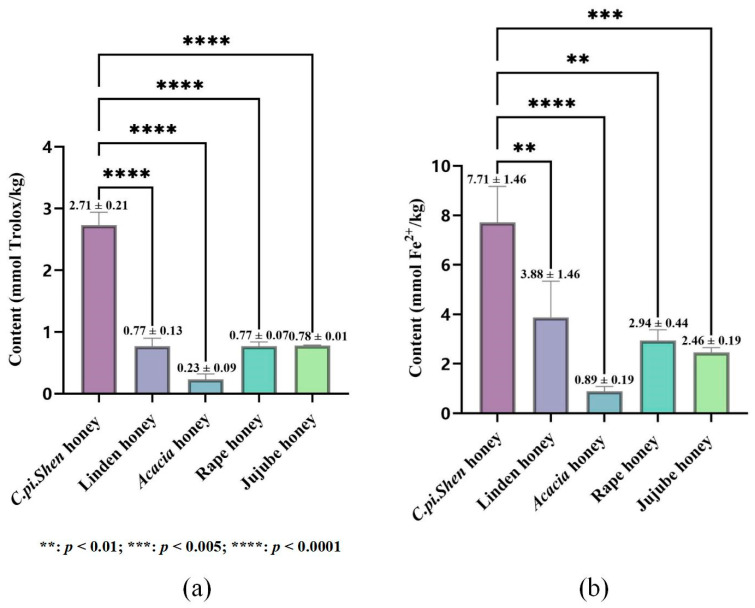
Results of (**a**) FRAP assay and (**b**) ABTS assay determination of *C. pi. Shen* honey and four other kinds of honey.

The presence of the transition metal ion Fe^2+^ has been demonstrated to readily instigate the Fenton reaction, which in turn produces hydroxyl radicals and accelerates the oxidation process. The addition of an antioxidant at this juncture results in its binding to Fe^2+^ and the subsequent inhibition of the oxidation reaction [42]. Therefore, the concentration of ferrous ions can indirectly reflect the strength of the antioxidant ability of honey. The measured contents of ferrous ions in five types of honey are shown in Figure 3a. *C. pi. Shen* honey demonstrated the highest FRAP content at 7.71 ± 1.46 mmol Fe^2+^/kg. Previous studies have shown [43] that antioxidant activity is directly proportional to the number of phenolic hydroxyl groups. Flavonoids (e.g., luteolin, quercetin, and kaempferol) contain a high number of phenolic hydroxyl groups, so it can be speculated that honeys containing more phenolic hydroxyl groups may have higher ferrous ion levels. As shown in Figure 2, kaempferol is one of the main phenolic compounds in *C. pi. Shen* honey, whereas *Acacia* honey contains less of these kinds of phenolic substances, which may be the reason why *C. pi. Shen* honey has a significantly higher ferrous ion content and antioxidant capacity than those of the other tested honeys.

#### 2.2.2. ABTS Free Radical Capacity Analysis

ABTS has been observed to react readily with potassium persulfate, undergoing oxidation to generate an ABTS radical cation ABTS^+^, which is a blue–green chromophore. The presence of antioxidants has been shown to inhibit the generation reaction of ABTS^+^ [44]. The primary antioxidant activity mechanism is based on single electron transfer [45]. The specific ABTS contents of the five honeys are depicted in Figure 3b. *C. pi. Shen* honey exhibits the highest ABTS activity, with a content of 2.73 ± 0.21 mmol Trolox/kg. Another study found that protocatechuic acid, *p*-hydroxybenzoic acid, and ellagic acid exert their antioxidant activity through electron transfer, hydrogen transfer, and free radical adduct formation [46,47]. This may be attributed to the fact that *C. pi. Shen* honey contains a markedly higher concentration of these components than other honeys, which results in a relatively high ABTS activity.

#### 2.2.3. DPPH Free Radical Scavenging Ability Analysis

**Figure 4:**
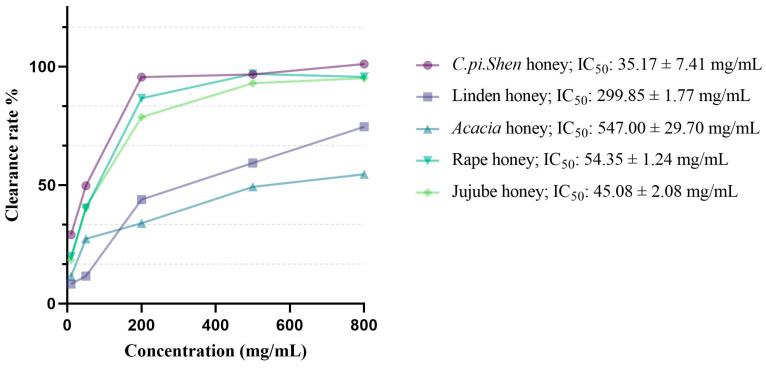
Results of the DPPH assay of *C. pi. Shen* honey and four other types of honey.

The DPPH free radical is a stable nitrogen-centered free radical, which prevents its dimerization. DPPH reacts with antioxidants, which reduces its deep purple color [32,48]. The lower the DPPH free radical scavenging rate IC_50_ value, the stronger the free radical scavenging ability [49]. The results of the determination of the DPPH free radical scavenging ability of the five tested honeys are shown in Figure 4, with the lowest DPPH free radical scavenging rate IC_50_ value of 35.17 ± 7.41 mg/mL observed in *C. pi. Shen* honey. This may relate to phenolic acids such as gallic acid or flavanols (a subtype of flavonoids) such as quercetin, kaempferol, and galangin. The authors of [50] explained that the scavenging activity of phenolic acids depends on the number and position of hydroxyl (-OH) groups and methoxy (-OCH_3_) substituents in the molecules. Moreover, gallic acid has been proven to possess the strongest radical scavenging activity among all the tested phenolic acids, and flavonoids such as quercetin also have a very high ability to scavenge DPPH radicals [50].

### 2.3. Correlation Analysis

Considering the antioxidant effect of phenolic acids in honey and combining the above results, it can be concluded that *C. pi. Shen* honey possesses a higher antioxidant capacity. The possibility of a correlation between the phenolic content and antioxidant activity of *C. pi. Shen* honey was analyzed using the Mantel test. In the Mantel test, the larger the correlation coefficient and the smaller the *p*-value, the greater the contribution of phenolic compounds to antioxidant activity.

**Figure 5:**
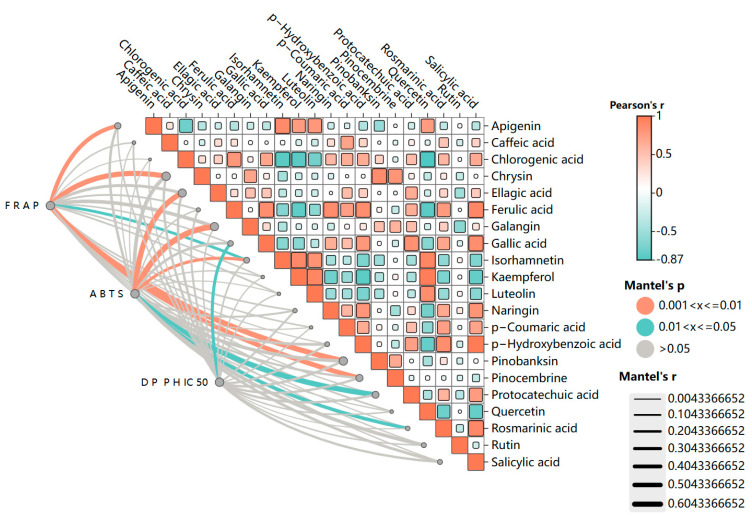
Correlation between the antioxidant activity and the contents of individual phenolic compounds of *C. pi. Shen* honey.

The results of the Mantel test are presented in Figure 5. A correlation was observed among the individual phenolic compounds present in *C. pi. Shen* honey. In particular, a number of phenolic acid compounds, including gallic acid, protocatechuic acid, chlorogenic acid, *p*-hydroxybenzoic acid, ferulic acid, rosmarinic acid, *p*-coumaric acid, and salicylic acid demonstrate a significant positive correlation with one another (*p* < 0.05), which is in accordance with the findings observed in the case of Kazakhstan buckwheat honey [16]. This finding corroborates the hypothesis that the contents of phenolic compounds in honey are interrelated. Significant positive correlations (*p* < 0.05) were also observed among the flavonoid components, including luteolin, quercetin, apigenin, kaempferol, and isorhamnetin.

The correlation analysis of the three antioxidant activities and the phenolic compounds of *C. pi. Shen* honey revealed that specific phenolic compounds are associated with the antioxidant activities of *C. pi. Shen* honey. The results demonstrate that apigenin, chrysin, pinobanksin, pinocembrine, and isorhamnetin are significantly correlated with FRAP activity (r > 0.5, *p* < 0.05). However, the concentrations of pinobanksin, chrysin, and pinocembrine were relatively low and represented a minor proportion of the phenolic constituents of *C. pi. Shen* honey (illustrated in Figure 2). Therefore, it may be posited that these flavonoid components exert a synergistic effect that results in the high FRAP activity of *C. pi. Shen* honey. The ABTS activity is significantly correlated with the contents of ellagic acid, galangin, isorhamnetin, protocatechuic acid, and rosmarinic acid (r > 0.5, *p* < 0.01). Among these potential phenolic-related compounds, only gallic acid is significantly correlated with the DPPH free radical scavenging activity of *C. pi. Shen* honey (r > 0.5, *p* < 0.05). This confirms the galloylation of gallic acid mentioned in previous studies [44], which means that the pyrogallic acid moiety of 2″-O-galloyl in *C. pi. Shen* honey may provide additional hydroxyl groups that provide *C. pi. Shen* honey with the ability to scavenge DPPH free radicals.

## 3. Materials and Methods

### 3.1. Materials

#### 3.1.1. Sample Information

The *C. pi. Shen* (*Codonopsis pilosula* (Franch.) var. modesta (*Nannf.*) L. T. Shen) honey samples were collected in August in Gansu, China. The *Acacia* (*Robinia pseudoacacia* L.) honey samples were collected in July in Liaoning, China. The Linden (*Tilia tuan Szyszyl.*) honey samples were collected in August in Shaanxi, China. The Rape (*Brassica napus* L.) honey samples were collected in March in Zhejiang, China. The jujube (*Ziziphus jujuba Mill.*) honey samples were collected in July in Liaoning, China. All samples were authenticated, and their physical properties are shown in Table 3. The samples were stored at 4 °C until analysis.

#### 3.1.2. Chemicals

1,1-Diphenyl-2-tri-nitrophenylhydrazine (DPPH), apigenin, caffeic acid, caffeic acid phenethyl ester (CAPE), chlorogenic acid, chrysin, ellagic acid, ferulic acid, galangin, gallic acid, isorhamnetin, kaempferol, luteolin, morin, naringin, *p*-coumaric acid, *p*-hydroxybenzoic acid, pinobanksin, pinocembrine, protocatechuic acid, quercetin, rosmarinic acid, rutin, salicylic acid (purity all ≥ 98.0%), and Folin–Ciocalteu reagent (1 mol/L) were all supplied by Shanghai Yuanye Bio-Technology company, Shanghai, China. The Iron Ion Reduction Reagent kit was sourced from Beijing Solarbio Technology company, Beijing, China. A SHIMSEN Styra MAX HLB Solid Phase Extraction Column (150 mg/6 mL) was supplied by Shimadzu company, Tokyo, Japan. The Total Antioxidant Capacity assay kit was provided by Nanjing Jiancheng Bioengineering Institute, Nanjing, China. All reagents used were of analytical or chromatographic purity.

### 3.2. Methods

#### 3.2.1. Melissopalynology Analysis

The melissopalynology analysis procedure was completed as follows: Ten grams of honey sample (accurate to 0.1 g) were weighed and placed in a centrifuge tube. Then, 20 mL of ultrapure water was added at 20 °C and mixed thoroughly. Next, the mixture was centrifuged at 8000 rpm for 10 min, the supernatant was carefully decanted, and another 20 mL of ultrapure water was added at 20 °C. Then, it was centrifuged again at 8000 rpm for 5 min. Finally, the upper liquid layer was gently poured off, and the predominant pollen types were determined under the SU8010 electron microscope (Hitachi, Tokyo, Japan) operated at 10.0 kV.

#### 3.2.2. Water Contents

Honey samples (2.0 g) were weighed and placed in a water bath at 45 °C until they melted. Subsequently, a small quantity of the honey sample was transferred into the refractometer, and the refractive index was measured. For temperatures above 20 °C, the factor was increased by 0.00023/1 °C, and for temperatures below 20 °C, it was reduced in a similar manner. Subsequently, the water content was determined by referring to the table provided in the Regulation. The results are expressed in percent.

#### 3.2.3. Color Measurement

The liquid honey was loaded into the measuring tube, the color was compared with standards, and the results obtained are expressed as mm Pfund.

#### 3.2.4. Phenolic Compound Determination

##### Total Phenolic Acid Content Determination

The total phenolic acid content was quantified using the Folin–Ciocalteu method [51]. An amount of 1 mL of sample solution (0.1 g/mL) was mixed with 1 mL of Folin–Ciocalteu reagent, followed by the addition of 5 mL of 1 mol/L Na_2_CO_3_. The mixture was diluted to 10 mL with ultrapure water, mixed, and then incubated at ambient temperature in the dark for 1 h. An amount of 200 µL of the mixture was transferred to a 96-well plate, and the absorbance at a wavelength of 760 nm was measured. The results were expressed as milligrams of protocatechuic acid equivalents per kg of honey (mg PCA/kg).

##### Total Flavonoid Content Determination

The total flavonoid content was determined according to the method of A. Arvouet-Grand et al. [52]. The sample was diluted with ultrapure water to 0.4 g/mL and centrifuged at 8000 r/min for 5 min. An amount of 3 mL of the supernatant was transferred to a 10 mL volumetric flask, and 1 mL of 1% AlCl_3_ (prepared with 95% ethanol) was added. The mixture was filled up to the mark with 95% ethanol and mixed well. The solution was left to stand at ambient temperature for 10 min. Finally, an amount of 200 µL of the solution was transferred to a 96-well plate, and the absorbance was measured at 405 nm. The results were expressed as milligrams of quercitrin equivalents per kg of honey (mg QUE/kg).

##### 23 Phenolic Compound Determination

An amount of 2.0 g of the sample was weighed and dissolved in 5 mL of ultrapure water, and the pH was adjusted to 6.0 with NH_3_·H_2_O. The sample was passed through the Styra MAX HLBV solid-phase extraction column activated with 5 mL of methanol and 5 mL of water, washed with 5 mL of water, dried, and then eluted with 5 mL of a 5% formic acid–methanol–acetonitrile (methanol/acetonitrile = 1:1) solution. The eluate was dried under nitrogen to near dryness at 40 °C and then dissolved in 1 mL of a 0.1% formic acid–80% methanol aqueous solution. Next, the mixture was passed through a 0.22 µm nylon filter and, finally, subjected to UPLC-MS/MS analysis.

The chromatographic conditions were as follows: chromatography column: ACQUITY UPLC HSS T3 (2.1 mm × 100 mm, 1.8 µm) (Waters, Milford, MA, USA); column temperature: 35 °C; injection volume: 2 µL; flow rate: 0.35 mL/min; mobile phase A: 0.1% formic acid water; mobile phase B: acetonitrile; and gradient elution program: 0~1 min, 5% B; 1~8.5 min, 5~70% B; 8.5~8.6 min, 70~100% B; 8.6~11 min, 100~5% B; and 11~14 min, 5% B.

The mass spectrometric conditions were as follows: electrospray ionization negative ion mode (ESI^−^); multiple reaction monitoring mode (MRM); gas temperature: 290 °C; gas flow: 11 L/min; sheath gas temperature: 350 °C; sheath gas flow: 9 L/min; nebulizer: 310 kPa; fragmentor: 380 V; and capillary: 3500 V. Mass spectrum analysis parameters of 23 phenolic and flavonoid compounds are shown in the Supplementary Materials (Table S1). The specific information of the Linear equation, R^2^, and quantification limit (LOQ) are presented in the Supplementary Materials (Table S2).

#### 3.2.5. Antioxidant Activity Evaluation

##### FRAP Assay Evaluation

The honey sample was assessed for its antioxidant capacity determination using the ferric reduction antioxidant power kit (Total Antioxidant Capacity assay kit, Solarbio, China). The analysis was conducted in accordance with the manufacturer’s indicated procedure as follows: Ten milligrams of FeSO_4_·7H_2_O were weighed and added to 0.9 mL of ultrapure water and 20 µL of concentrated FeSO_4_ to prepare a 40 μmol/mL standard solution. This standard solution was diluted with ultrapure water to obtain standard working solutions of 0.15, 0.1, 0.05, 0.025, 0.00625, 0.003125, and 0.00156 μmol/mL. Then, 100 μL of the FeSO_4_ standard working solution (with ultrapure water as the blank) was thoroughly mixed with 100 μL of Reagent No. 2 in the kit. This was left to react for 10 min, and then the absorbance was measured at 593 nm. A standard curve was established based on the final concentration of Fe^2+^ (μmol/mL) and the corresponding absorbance. Next, 6 μL of the 0.5 g/mL sample was added to 180 μL of the mixture in the kit and 18 μL of ultrapure water and thoroughly reacted for 10 min. Then, 200 µL of the mixture was absorbed and placed in a 96-well plate. Then, the absorbance was measured at 593 nm. Finally, the ferric ion reducing capacity was calculated based on the standard curve. The unit is expressed as mmol Fe^2+^/kg honey.

##### ABTS Free Radical Capacity Evaluation

The ABTS assay was carried out in accordance with the ABTS method described in the kit (Total Antioxidant Capacity assay kit, Nanjing Jiancheng Bioengineering Institute, China). A 10 mmol/L Trolox standard solution was diluted with ultrapure water to produce 0.1, 0.2, 0.4, 0.8, and 1.0 mmol/L standard working solutions. An amount of 10 µL of Trolox standard working solution of different concentrations (10 µL ultrapure water as a blank control; the sample was diluted to 0.50 g/mL) was mixed with 20 µL of peroxidase solution and 170 µL of ABTS working solution and incubated for 6 min at ambient temperature. The absorbance was then measured at 405 nm. The ABTS radical scavenging rate was calculated based on the standard curve and expressed in mmol Trolox/kg honey.

##### DPPH Free Radical Scavenging Ability Evaluation

The DPPH radical scavenging test is based on the method of Larrauri J A. [53] et al. with slight modifications. The procedure was conducted as follows: DPPH standard working solution was prepared at a concentration of 0.1 mmol/L with ethanol and diluted with ultrapure water to concentrations of 0.01, 0.05, 0.20, 0.50, and 0.80 g/mL. Then, 100 µL of the different concentrations of sample solutions and water were added into 96-well plates, and 100 µL of the DPPH standard working solution was added. This was mixed thoroughly and incubated at room temperature in the dark for 30 min. Absorbance was measured at 517 nm wavelength. The DPPH radical scavenging ability (%) was calculated as follows:

$$\%=\frac{A_{1}-\left(A_{2}-A_{3}\right)}{A_{1}}\times 100\%$$

In the equation, *A*_1_ represents the absorbance value after mixing 100 µL of water and 100 µL of DPPH standard working solution; *A*_2_ represents the absorbance value after mixing 100 µL of different concentrations of honey samples and 100 µL of DPPH standard working solution; and *A*_3_ represents the absorbance value after mixing 100 µL of different concentrations of honey samples and 100 µL of anhydrous ethanol.

### 3.3. Statistical Analysis

The SPSS version 26 software for Windows was used for statistical analysis. Three parallel determinations were performed for each sample and expressed as mean ± standard. The Tukey test was employed to evaluate the significance of differences among the mean values of the analyzed features, and the graphs were generated using GraphPad Prism version 9.0 software. The correlation analysis used the Mantel test, and the graphs were drawn using the online website Chiplot (https://www.chiplot.online (accessed on 16 October 2024)).

## 4. Conclusions

This study aimed to investigate the phenolic composition and in vitro antioxidant activity of *C. pi. Shen* honey from Gansu Province of China, with reference to the results of *Acacia* honey, linden honey, rape honey, and jujube honey. The findings indicated that *C. pi. Shen* honey is characterized by a high concentration of phenolic acids and flavonoids and displays considerable antioxidant activity. Further quantitative analysis of 23 phenolic compounds revealed that protocatechuic acid and kaempferol were the higher phenolic compounds in *C. pi. Shen* honey, 1855.6 ± 640.7 μg/kg and 1353.9 ± 815.8 μg/kg, respectively. Correlation analysis showed that different phenolic compounds in *C. pi. Shen* honey contributed to antioxidant activity to different degrees. However, traditional in vitro antioxidant assays still have some limitations as they either do not detect or poorly detect reaction kinetics and are non-specific for antioxidants. Therefore, a more detailed discussion of the actual bioavailability of these antioxidant components in honey will be more conducive to understanding their actual biological potential, which is the aim of our next stage of research. This study provides data to support the evaluation of the nutritional functional components of *C. pi. Shen* honey.

## Figures and Tables

**Table 1:** Phenolic compound contents in *C. pi. Shen* honey and four other kinds of honey (μg/kg).

No.	Phenolic Compounds	*C. pi. Shen* Honey	Linden Honey	*Acacia* Honey	Rape Honey	Jujube Honey
1	Apigenin	118.7 ± 46.0 ^b^	<2.5	<2.5	<2.5	268.2 ± 143.3 ^a^
2	Caffeic acid	122.6 ± 27.0 ^c^	339.7 ± 42.8 ^b^	48.2 ± 29.5 ^c^	35.9 ± 12.1 ^c^	722.3 ± 320.9 ^a^
3	CAPE	<2	<2	36.0 ± 3.5 ^a^	7.4 ± 1.2 ^c^	17.8 ± 14.5 ^b^
4	Chlorogenic acid	337.2 ± 154.3 ^b^	<5	<5	28.0 ± 0.6 ^b^	2654.8 ± 815.2 ^a^
5	Chrysin	9.1 ± 7.3 ^c^	140.8 ± 10.2 ^b^	274.1 ± 42.4 ^a^	111.3 ± 5.0 ^b^	342.2 ± 147.9 ^a^
6	Ellagic acid	43.6 ± 10.9 ^b^	276.6 ± 2.6	13.0 ± 0.1 ^c^	230.8 ± 9.4 ^a^	17.3 ± 2.9 ^c^
7	Ferulic acid	29.5 ± 14.5 ^c^	74.7 ± 2.0 ^a^	33.4 ± 1.6 ^b,c^	16.8 ± 0.3 ^c^	62.9 ± 28.3 ^a,b^
8	Galangin	5.9 ± 4.6 ^c^	28.3 ± 1.9 ^c^	201.1 ± 37.1 ^a^	83.3 ± 10.2 ^b^	208.3 ± 91.5 ^a^
9	Gallic acid	217.8 ± 73.5 ^b^	139.0 ± 125.9 ^c^	32.7 ± 1.3 ^c^	400.3 ± 67.0 ^a^	54.2 ± 12.8 ^c^
10	Isorhamnetin	758.7 ± 316.3 ^a^	89.6 ± 48.5 ^b^	53.4 ± 46.1 ^b^	141.2 ± 20.5 ^b^	45.5 ± 30.8 ^b^
12	Kaempferol	1353.9 ± 815.8 ^a^	207.7 ± 0.7 ^b^	85.3 ± 62.0 ^b^	265.3 ± 38.4 ^c^	300.4 ± 78.3 ^c^
11	Luteolin	95.2 ± 69.6 ^a,b^	77.3 ± 9.3 ^a,b^	3.4 ± 2.4 ^b^	9.5 ± 1.2 ^b^	187.1 ± 109.7 ^a^
13	Morin	<5	<5	<5	<5	<5
14	Naringin	12.0 ± 2.3 ^a,b^	10.2 ± 0.1 ^b^	10.4 ± 0.4 ^a,b^	15.3 ± 1.3 ^a^	10.6 ± 0.1 ^a,b^
15	*p*-Coumaric acid	54.4 ± 12.7 ^b^	137.1 ± 43.4 ^a,b^	32.7 ± 27.2 ^c^	25.3 ± 1.8 ^c^	211.0 ± 149.0 ^a^
16	*p*-Hydroxybenzoic acid	145.1 ± 78.6 ^a^	85.4 ± 18.0 ^a^	63.2 ± 46.4 ^a^	108.9 ± 6.5 ^a^	50.0 ± 8.5 ^a^
17	Pinobanksin	6.0 ± 2.8 ^d^	387.7 ± 139.8 ^b,c^	544.8 ± 100.7 ^b^	185.3 ± 21.2 ^c,d^	870.1 ± 434.4 ^a^
18	Pinocembrine	4.6 ± 1.6 ^c^	223.5 ± 33.7 ^b^	475.4 ± 120.5 ^a^	121.5 ± 7.4 ^b, c^	444.7 ± 331.0 ^a^
19	Protocatechuic acid	1855.6 ± 640.7 ^a^	724.2 ± 22.3 ^b^	23.9 ± 5.6 ^b^	322.3 ± 43.5 ^b^	322.0 ± 111.4 ^b^
20	Quercetin	573.9 ± 302.7 ^a^	73.3 ± 0.1 ^a^	<2	124.0 ± 46.0 ^a^	22.0 ± 15.9 ^a^
21	Rosmarinic acid	15.0 ± 2.1 ^a^	12.2 ± 0.1 ^a^	12.3 ± 0.1 ^a^	12.3 ± 0.1 ^a^	13.9 ± 2.3 ^a^
22	Rutin	55.0 ± 8.50 ^a^	28.8 ± 2.7 ^b^	15.8 ± 0.9 ^c^	43.5 ± 23.1 ^a,b^	41.1 ± 32.1 ^a,b^
23	Salicylic acid	144.6 ± 80.0 ^a^	90.6 ± 12.6 ^a^	63.5 ± 44.6 ^a^	110.6 ± 0.2 ^a^	54.4 ± 12.0 ^a^

a, b, c, d: the grouping of information for the significance test.

**Table 2:** Phenolic compounds determined in *Codonopsis* genus honeys from different origins and other information.

Botanic Origin	Geographical Origin	Number of Phenolic Compounds Investigated	Top 2 Compounds and Mean Contents (μg/kg)	Reference
*C. pi. Shen*	Gansu, China	23	Protocatechuic acid (1855.6)	/
			Kaempferol (1353.9 ^a^)	
*Codonopsis 1*	Xinjiang, China	18	Benzoic acid (611)	[35]
			*p*-Hydroxybenzoic acid (513)	
*Codonopsis 2*	Jinlin, China	38	*p*-Hydroxybenzoic acid (924.2) Kaempferol (637.9 ^b^)	[36]

a, b: the grouping of information for the significance test.

**Table 3:** Physical properties of *C. pi. Shen* honey and four other kinds of honey.

	*C. pi. Shen* Honey	*Acacia* Honey	Linden Honey	Rape Honey	Jujube Honey
Color Scale (mm Pfund)	88~117	32~40	53~63	93~105	90~110
Water (%)	16.5~17.8	16.2~17.0	16.5~17.0	16.6~17.2	16.7~17.7

## Data Availability

The data presented in this study are available in the article and the Supplementary Materials.

## Supplementary Materials

The following supporting information can be downloaded at: https://www.mdpi.com/article/10.3390/molecules30020370/s1: Table S1: Mass spectrum analysis parameters of 23 phenolic and flavonoid compounds; Table S2: Linear equations of phenolic compounds, R^2^, and limit of quantification (LOQ).
