# Supplementary material for: Phenolic Acid and Flavonoid Content Analysis with Antioxidant Activity Assessment in Chinese C. pi. Shen Honey
Source: Molecules. 2025 Jan 17;30(2):370. doi: 10.3390/molecules30020370 (PMC11767644; doi:10.3390/molecules30020370)
Supplement: Supplementary file 1 [file molecules-30-00370-s001.zip › molecules-3404277-supplementary.pdf]

Table S1. Mass spectrum analysis parameters of 23 phenolic and flavonoid compounds.

No.	Phenolic Compounds	Retention time (min)	Precursor Ion (m/z)	Product Ion (m/z)	Collision Energy (V)
1	Apigenin	6.985	269	117*<br>151	30<br>24
2	Caffeic acid	4.414	180	136*<br>118	15<br>25
3	CAPE	8.516	283.1	135*<br>179	20<br>12
4	Chlorogrnic acid	3.958	353.1	353.1*<br>190.9	15<br>15
5	Chrysin	8.378	253.1	63*<br>143	32<br>24
6	Ellagic acid	4.898	301	145*<br>229	25<br>38
7	Ferulic acid	5.255	193.1	134*<br>177.9	15<br>15
8	Galangin	8.536	269	269*<br>169	24<br>24
9	Gallic acid	7.205	169	125*<br>79	15<br>19
10	Isorhamnetin	7.211	315.2	300.1*<br>227.4	22<br>32
11	Kaempferol	7.103	285	93*<br>257	20<br>20
12	Iuteolin	6.392	285.1	133*<br>150.9	40<br>30
13	Morin	6.190	301	151*<br>125	16<br>16
14	Naringin	5.218	579	271*<br>459	37<br>25
15	*p*-Coumaric acid	5.056	163	119*<br>93	15<br>15
16	*p* -Hydroxybenzoic acid	6.108	137	137*<br>93	10<br>10
17	Pinobanksin	7.205	271	252.9*<br>197	22<br>28
18	Pinocembrine	8.487	255.1	151*<br>213	16<br>12
19	Protocatechuic acid	3.475	153	190*<br>81	15<br>20
20	Quercetin	6.441	301.1	151.1*<br>179	22<br>18
21	Rosmarinic acid	5.558	359	161*<br>197	12<br>15
22	Rutin	4.757	609	301*<br>271	40<br>58
23	Salicylic acid	6.108	137.1	93.1*<br>137.1	14<br>14

Table S2. Linear equations of phenolic compounds, R^2, and limit of quantification (LOQ).

NO.	Phenolic Compound	Linear equation	R^2	LOQ（μg/kg）
1	Apigenin	Y=6271.98x+149449.95	0.9995	2.5
2	Caffeic acid	Y=502.60x+3818.06	0.9995	10
3	CAPE	Y=12154.89x+2344.45	0.9998	2
4	Chlorogrnic acid	Y=2339.59x-4877.84	0.9998	5
5	Chrysin	Y=2090.15x	0.9997	5
6	Ellagic acid	Y=212.89x-7895.32	0.9996	5
7	Ferulic acid	Y=362.02x-6187.68	0.9997	5
8	Galangin	Y=584.18x	0.9992	5
9	Gallic acid	Y= 645.74x-26057.02	0.9995	5
10	Isorhamnetin	Y=11404.28x+110358.74	0.9999	2
11	Iuteolin	Y=6607.62x	0.9999	2
12	Kaempferol	Y=491.71x+3557.90	0.9998	10
13	Morin	Y=2478.38x-46596	0.9991	5
14	Naringin	Y=642.00x-12791.90	0.9991	2
15	*p*-coumaric acid	Y=3285.66x+34901.30	0.9997	5
16	*p* -Hydroxybenzoic acid	Y=3671.822775x+41988.16	0.9993	5
17	pinobanksin	Y=6740.70x+4536.11	0.9997	2
18	Pinocembrine	Y=990.81x-13449.20	0.9998	2
19	Protocatechuic acid	Y=2252.43x	0.9995	2.5
20	Quercetin	Y=22823.76x+372608.84	0.9993	2
21	Rosmarinic acid	Y =1867.186127x-33557.26	0.9998	5
22	Rutin	Y=508.50x-5629.28	0.9999	5
23	Salicylic acid	Y=4593.54x+25502.80	0.9998	5
